# Letters to the Editor / Author’s reply

**DOI:** 10.14744/nci.2022.98958

**Published:** 2022-02-18

**Authors:** Mikail Ozdemir, Can Ilgin, Melda Karavus, Seyhan Hidiroglu, Nimet Emel Luleci, Pinar Ay, Abdullah Sarioz, Dilsad Save

**Affiliations:** 1 Oguzeli District Health Center, Turkish Ministry of Health, Gaziantep, Turkey; 2 Department of Public Health, Marmara University Faculty of Medicine, Istanbul, Turkey; 3 Edremit Province Health Directory, Turkish Ministry of Health, Van, Turkey

To the Editor,

We would like to acknowledge Ozgur and Eser for their valuable comments regarding our article entitled “Adaptation of the knowledge about childhood autism among health workers questionnaire: Turkish version” [1, 2]. In their letter, Ozgur and Eser [2] indicate that removing some items from the original scale during adaptation might impair the content validity and thus should be avoided. While this view is legitimate, we should be aware that it also has some exceptions. Modifications in validated scales might be needed due to the developments in the scientific field and also the changing needs in a community. The scale that is the subject of this discussion was developed by Bakare et al. [3] in Nigeria in 2008, and its adaptation to our community certainly required some modifications. As is known, autism spectrum disorder and childhood-onset schizophrenia have overlapping symptomatology, but currently are defined as two separate entities [4]. We should also note that individuals with autism spectrum disorder might have a range of IQ, varying from low to above average [5]. The link of autism to autoimmunity is still questionable and warrants further evidence [6]. Hence, the fourth domain focusing on autoimmunity as a cause, and identifying autism as childhood schizophrenia and as a form of mental retardation might be misleading. Moreover, Ozgur and Eser [2] also draw attention to the insufficient internal consistency in the fourth domain and indicated that the scores of this domain should be evaluated with caution.

While developing scales, a special care should be taken not to stigmatize the affected individuals through the questions used. Autism is surrounded with huge stigma [7]. We were concerned that Domain 4 might lead to false information and even more stigmatizing attitudes, which also influenced our decision of removing this section.

Another item was removed from the questionnaire since; all participants provided full and correct answers to it.

The permission for the adaptation of the questionnaire was obtained from Dr. Muideen Bakare in 2015, who was the first author in the original validation study. Subsequently, we consulted Dr. Muideen Bakare for the removal and changes in the items and also received an approval for the new version.

Finally; researchers can decide which version of the scale to use, taking into consideration their objectives. A version which is useful for a group of researchers might not serve others. In this respect, we believe that an environment discussing the advantages and disadvantages of different versions would be more fruitful instead of advising which version to use.
